# Supplementary material for: The Idiopathic Pulmonary Fibrosis-Associated Single Nucleotide Polymorphism RS35705950 Is Transcribed in a MUC5B Promoter Associated Long Non-Coding RNA (AC061979.1)
Source: Noncoding RNA. 2022 Dec 8;8(6):83. doi: 10.3390/ncrna8060083 (PMC9781688; doi:10.3390/ncrna8060083)
Supplement: Supplementary file 1 [file ncrna-08-00083-s001.zip › ncrna-1975516-supplementary-for PUB/FigureS1.pdf]

CTGCTGCAAAGTCCATGCTACTGGAAGCCTCGAAGTAGGGGGGATTCTGT
TCTAGTCTTTGTCAAATCCCACTGCCCATGGCAGCACCAGGACCCAGTTG
GGGCTCCTTGGAACTGGCAGGAAGGAATCGGGTGGGGAGACAGGCAGAGA
AGGGGGTCTGTGCAAAGACCAGGAGAAACCAGAGACAGGTCGTGGCGGGG
GCTGAGACCTTCACACAGGGCAGGGGCCGCCCCGGGGGGTTCTCCTTGTC
TTGCAGCCCCTGTGCAGGGCATCCTCAGAGCAGGGGCAGCCCAGGGCACC
GGGACGCCCAGGTGGAAGGTGACCTGCCATCCTGCAGCTTCACTTCCTGC
CGGGTGATTCGGTACCCCTGGTTGTGCCTGTCGCTCAGTGGGCCAGGGTC
TAAGGGCTGTGAAGACTCAACATGCCCCCACCTGCTACTTCTGAACACCA
GGCACTGGCTCTGAGACCCCCGGGCCTTGCTGGACATCTCCCCAGGTGTA
CTGGGCCAGGGGACAGGGGCCTGGCCATCCCAACACCCAGGAGCAAGCAG
CCCGTCACCTGCCCAGGTCCCCGAGGCCTGGAACACCTTCCTGCTGGGCC
CACCCAGCCCTGGACCTGTCCCGCTTGGTCACACGATGGACCCTCGGCC
CATCAGCAGgtgagcccccaggagcgtgcgtctggcctggtaaggcctcc
accccaggagttgggggcccccgtgccagggagcaggaggctgccgagg
tggagggtcccacacagctaccactccctatccccagcacagcctggggc
ctggctctgagtacacatcctggggcctggctctgagcagaccaagagcc
catccctgctttgtgacccccctgggctgtgcctgacaccccaggtgtcca
gcgtggagctggggcccagctcagtgcctgggagctgatggaccctgggg
cccggctcagtgcctggtggctgatggacactggggcctggctcaaacct
gcaccgctgtggtcgggggaggggagggctgagccacgtggggaccccag
ccccagtgacgactctttgcggtggccaagccctccaggtgtcccccagg
gctgagggctgggcttggggcagctggtgacagcagatggtggccctga
tcactggtgcctggacggcctctgaaggggtctgtggggtcctggacggg
tccccattcatggcaggattaaccccccctcgggttctgtgtggtctaggc
cgcccctttgtctccactgcccccctggccagaatgagggacagtgaccca
cccagggctgggcctggctcagactccgtcagagccgcagggcaagttcc
tggcacgtccgaggtgggaggctcctctgcgctccaggaggctgtgcctg
gcccccccttcccggcaggaaccggctgtgtccctttccttcctttatctt
ctgttttcagCGCCTTCAACTGTGAAGAGGTGAACTCTTCAAACACGCTG
AGCAAACAGGCCCGACTCCCAGGGCCGCATCCGGGATGTCTCAATAGCTG
TGGCCTTGACGTCCACCTCGGACCCCTGCCCCGGACCCAGCCCAGTTCCC
AATGGGCCCTCTGCCCGGGGAGGTGCCTAGTGGGAGGGACGAGGGCAAAG
TCGGGGCCCCCACTTGTTTGGTGTCACTGTGTGCCAGCGGCCACTGGCGG
GCGAGGCTGTTCC

Figure S1: **AC061979.1 - DNA Sequence.** Uper case letters - exons; lower case letters - intron; green highlight - SMAD2/3 binding motif; pink highlight - FOXA2 binding motif.
