# Supplementary material for: The Idiopathic Pulmonary Fibrosis-Associated Single Nucleotide Polymorphism RS35705950 Is Transcribed in a MUC5B Promoter Associated Long Non-Coding RNA (AC061979.1)
Source: Noncoding RNA. 2022 Dec 8;8(6):83. doi: 10.3390/ncrna8060083 (PMC9781688; doi:10.3390/ncrna8060083)
Supplement: Supplementary file 1 [file ncrna-08-00083-s001.zip › ncrna-1975516-supplementary-for PUB/FigureS2.pdf]

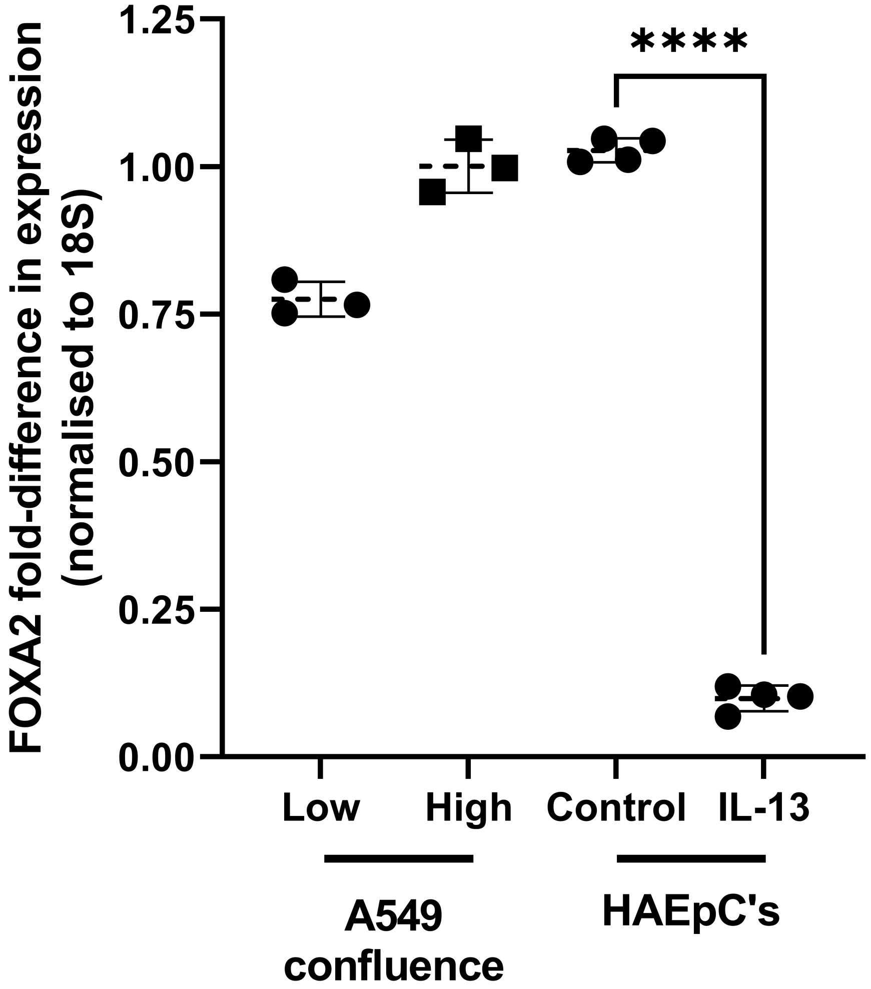

Figure S2: **FOXA2 expression levels in A549 and HAEpC's.** The levels of FOXA2 mRNA were determined in A549 cells at low and high confluence levels and HAEpC's with and without IL-13 stimulation. Expression, normalised to 18S rRNA levels, was calculated relative to high confluence A549s or paired unstimulated HAEpC's. ****: $p<0.0001$.
